# Development of High-Efficiency, Magnetically Separable Palladium-Decorated Manganese-Ferrite Catalyst for Nitrobenzene Hydrogenation

**DOI:** 10.3390/ijms23126535

**Published:** 2022-06-10

**Authors:** Viktória Hajdu, Gábor Muránszky, Miklós Nagy, Erika Kopcsik, Ferenc Kristály, Béla Fiser, Béla Viskolcz, László Vanyorek

**Affiliations:** 1 Institute of Chemistry, University of Miskolc, Miskolc-Egyetemváros, 3515 Miskolc, Hungary; kemviki@uni-miskolc.hu (V.H.); kemmug@uni-miskolc.hu (G.M.); ria.toth1@gmail.com (E.K.); kemfiser@uni-miskolc.hu (B.F.); bela.viskolcz@uni-miskolc.hu (B.V.); 2 Institute of Mineralogy and Geology, University of Miskolc, Miskolc-Egyetemváros, 3515 Miskolc, Hungary; askkf@uni-miskolc.hu

**Keywords:** manganese ferrite, spinel, aniline, nanoparticles, Pd/MnFe_2_O_4_, nitrobenzene

## Abstract

Aniline (AN) is one of the most important compounds in the chemical industry and is prepared by the catalytic hydrogenation of nitrobenzene (NB). The development of novel, multifunctional catalysts which are easily recoverable from the reaction mixture is, therefore, of paramount importance. Compared to conventional filtration, magnetic separation is favored because it is cheaper and more facile. For satisfying these requirements, we developed manganese ferrite (MnFe_2_O_4_)–supported, magnetically separable palladium catalysts with high catalytic activity in the hydrogenation of nitrobenzene to aniline. In addition to high NB conversion and AN yield, remarkable aniline selectivity (above 96 n/n%) was achieved. Surprisingly, the magnetic support alone also shows moderate catalytic activity even without noble metals, and thus, up to 94 n/n% nitrobenzene conversion, along with 47 n/n% aniline yield, are attainable. After adding palladium nanoparticles to the support, the combined catalytic activity of the two nanomaterials yielded a fast, efficient, and highly selective catalyst. During the test of the Pd/MnFe_2_O_4_ catalyst in NB hydrogenation, no by-products were detected, and consequently, above 96 n/n% aniline yield and 96 n/n% selectivity were achieved. The activity of the Pd/MnFe_2_O_4_ catalyst was not particularly sensitive to the hydrogenation temperature, and reuse tests indicate its applicability in at least four cycles without regeneration. The remarkable catalytic activity and other favorable properties can make our catalyst potentially applicable to both NB hydrogenation and other similar or slightly different reactions.

## 1. Introduction

The global aniline market reached a volume of 8.93 million tons in 2021 and is expected to reach 12.47 million tons by 2027. This huge production volume is a clear indicator that aniline is one of the most important bulk chemicals, with wide applications in the manufacture of herbicides, dyes, pigments, pharmaceuticals, and polymers (e.g., polyurethanes, ~75% of world production) [1,2,3,4,5]. Aniline is produced industrially by the catalytic hydrogenation of nitrobenzene carried out either in the gas or liquid phase. In most cases, the liquid phase process is performed in an organic solvent, such as methanol [6], ethanol [7], or isopropanol [8]. Heterogeneous catalysts, such as Raney nickel [9], copper [10], gold [11], platinum [12], and palladium [13], are commonly used in the hydrogenation of nitrobenzene. Recently, a number of palladium-based catalysts have been developed and used in industrial processes due to their high activity and selectivity. Recovery of homogeneous Pd catalysts from the reaction medium is cumbersome, time-consuming, and limits recycling; therefore, the development of heterogeneous catalysts has received much attention [14]. Among the various substrates, clays with high thermal and mechanical stability were one of the most facile [15]. Large specific surface area (a = A/V, m^2^/m^3^) is a key factor in heterogeneous catalysis since the reaction happens on the catalyst surface. Larger surface yields higher rate of reaction, hence the application of nanoparticles is favored. Moreover, nanoparticles with different geometries can also be combined. For example placing palladium on poly-dopamine-decorated halloysite nanotubes hybridized with N-doped porous carbon monolayer (Pd@Hal-pDA-NPC) yielded a hybrid catalyst which proved to be very effective in C–C coupling (Sonogashira, Heck and Suzuki reactions) and the hydrogenation of nitro compounds with high recyclability [16]. The adhesion between the catalyst and the clay support can be further enhanced by decoration and support with various ligands, such as dendrimer (PAMAM) [17], different diamines [18], or multi-dentate ligands [19]. Instead of clay, natural polymers, such as ligand-decorated chitosan, can also be used as support [20]. Using these supports has the advantages of easy catalyst recovery and reusability without losing catalytic activity and with little Pd dissolution; however, they are difficult to make, and therefore cheaper alternatives, such as activated carbon, are still in use.

Palladium catalysts on activated carbon are the main types, although their separation from the reaction medium is difficult and time-consuming due to their very small (several 10–100 nm) particle size. The use of magnetic materials as catalyst supports is a novel approach in the hydrogenation of NB [21,22,23,24]. Magnetic materials allow easy and efficient separation of the catalyst using an external magnet or magnetic field, avoiding complicated and time-consuming additional separation operations such as filtration and centrifugation [25]. Spinel ferrites (MFe_2_O_4_, M = Mn, Co, Cu, Ni, etc.) appear as new and promising heterogeneous catalysts due to their ferromagnetic properties and stable mineral structures. They are widely used in information storage, ferrofluid technology, electronic devices, biomedical applications, and catalysis [26,27,28,29]. Manganese(II) ferrite (MnFe_2_O_4_) has an inverse spinel structure, high adsorption capacity, and exceptional chemical stability. MnFe_2_O_4_ nanoparticles can be prepared by a variety of methods, such as hydrothermal [30,31], solvothermal [32,33], coprecipitation [34,35], sol-gel [36], microemulsion [37], combustion [38], and sonochemical synthesis [39].

Sonochemical synthesis has a few advantages compared to conventional methods. Catalysts are generally prepared in several steps. During the activation step, metal ions (e.g., palladium ions), oxides, and complex ions are reduced with hydrogen gas to a catalytically active form, namely the metal phase; however, this reduction step is generally time- and energy-consuming. In contrast, using ultrasonic cavitation, the catalyst preparation process is simplified, and no post-activation in hydrogen is required as the precious metals are present in the elemental state on the support.

In our work, we used a simple process to prepare novel, magnetic MnFe_2_O_4_-supported Pd catalysts, the performance of which was tested in nitrobenzene hydrogenation at four different temperatures. Important catalytic properties, such as conversion, yield, selectivity, ease of separation (magnetically), and reusability were also investigated in detail.

## 2. Results and Discussion

### 2.1. Synthesis and Characterization of the Developed Magnetic Catalysts

For the economical production of aniline, the price of the catalyst is a key factor. One of the most facile and cheapest methods for the preparation of the magnetic support nanoparticles is sonochemical activation combined with combustion. During the first step, upon the action of ultrasound, highly dispersed metal hydroxide nanoparticles formed from the metal nitrate precursors in polyethylene-glycol-based dispersion. PEG400 was used in this study since it is a viscous liquid at room temperature, and it yielded the desired small (metal oxide/hydroxide) particle size. The chemical effects of ultrasound irradiation are, primarily, attributed to its acoustic cavitation: the formation, growth, and implosive collapse of bubbles in the irradiated liquid. During the collapse, there is a high energy density from the conversion of the kinetic energy of the liquid’s motion into the heating of the contents of the bubble, which can cover the energy requirements of the chemical reactions, such as the formation of metal hydroxides from their nitrate precursors in the polyol phase. Iron(III) was added to Mn(II) in a 2:1 molar ratio (16 mmol vs. 8 mmol) to obtain the favored spinel structure with the following formula: MFe_2_O_4_ (where M stands for Mn, Cd, Co, Ni, Cu, Zn, etc.). Deviation from the stoichiometric form of the spinel structure may result in the formation of undesirable, non-magnetic metal oxides.

**Figure 1:**
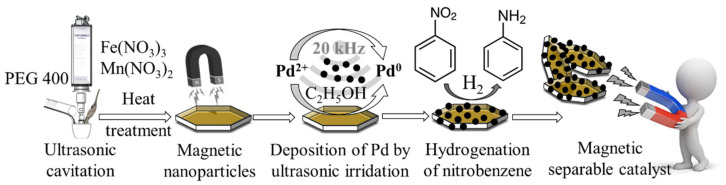
Scheme of the preparation and application of the magnetic Pd/MnFe_2_O_4_ catalyst.

In the second step, during the combustion method, the PEG-based colloid system of the iron and manganese hydroxides was heated in a furnace in the presence of an air atmosphere at 573 K, 623 K, and 673 K. The duration of the heat treatment was 3 h in each case. After burning of the PEG and the dehydration of the metal hydroxide nanoparticles, the expected spinel structures with magnetic properties formed. These MnFe_2_O_4_ samples were used as magnetic catalyst support for the preparation of palladium-decorated spinel catalysts. Palladium was also deposited on the surface of the support using a sonochemical step. During the process, elemental palladium nanoparticles formed from the Pd(II) ions and the alcohol (patosolv), which acted as the reducing agent. The synthesis, properties, and further application of the catalyst nanoparticles are summarized in Figure 1 and Table 1.

**Figure 2:**
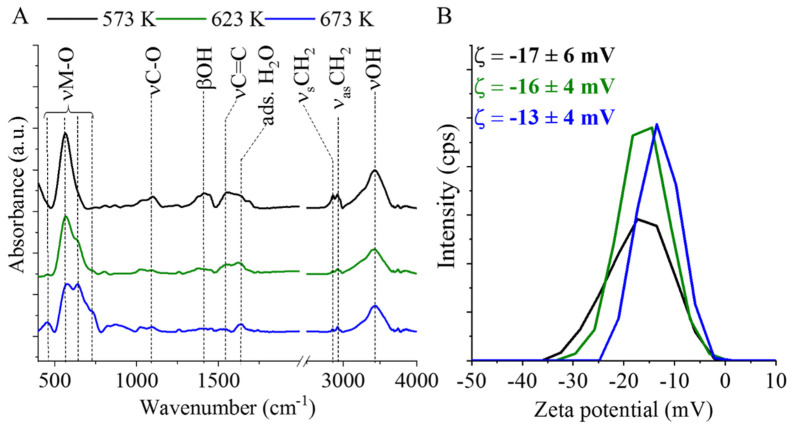
FTIR spectra (**A**) and Zeta potential distributions (**B**) of the magnetic catalyst supports.

Surface polarity and the presence of functional groups on the surface are important factors which determine the dispersibility of the nanoparticles in the liquid phase. Furthermore, the functional groups could serve as potential anchor points for the catalytically active metals on the supports. On the other hand, surface polarity, and the potential binding centra (functional groups, crystal defect sites etc.), play a great role in the adsorption and desorption processes of the reactant and product molecules during catalytic hydrogenation reactions. Henceforth, it is important to determine the presence or absence of certain functional groups and the corresponding Zeta potential of the system which contribute to the dispersibility and colloidal stability in water or aqueous solutions. Thus, FTIR and Zeta potential measurements were carried out in the case of the three magnetic catalyst supports which were prepared at different temperatures (Figure 2A,B).

In the case of the manganese ferrite support synthesized at 573 K, two absorption bands at 448 cm^−1^ and 568 cm^−1^ wavelengths were identified in the spectrum, possibly arising from the intrinsic stretching vibration modes of the metal–oxygen bond at the octahedral and tetrahedral sites of the MnFe_2_O_4_ [40]. Furthermore, based on the band at 1099 cm^−1^, C–O bonds are also located on the surface, indicating the presence of alcoholic, carbonyl, and carboxyl functional groups. The band at 1557 cm^−1^ can be associated with the νC=C vibration mode of the deposited carbon from the thermal decomposition of the polyol (PEG). Additional carbon vibration bands are also visible at 2864 cm^−1^ and 2927 cm^−1^, which correspond to the symmetric and asymmetric stretching vibration of the aliphatic and aromatic C–H bonds, respectively. The spectra of the samples synthesized at higher temperatures (623 K and 673 K) clearly differ as the intensity of νC–O, νC=C and νC–H bands decreased because of the partial oxidation of carbon as the heating occurred in the air atmosphere. The band at 1410 cm^−1^ corresponds to the βOH vibration mode. The presence of the –OH stretching vibration is further confirmed by a band at 3429 cm^−1^. Furthermore, adsorbed water molecules are also detected as an OH vibration band at 1641 cm^−1^. At higher temperatures, distinct shoulders can be found on the bands of the metal–oxygen vibrations at 642 cm^−1^ and 726 cm^−1^ due to the formation of a new phase, the magnetite beside MnFe_2_O_4_.

**Figure 3:**
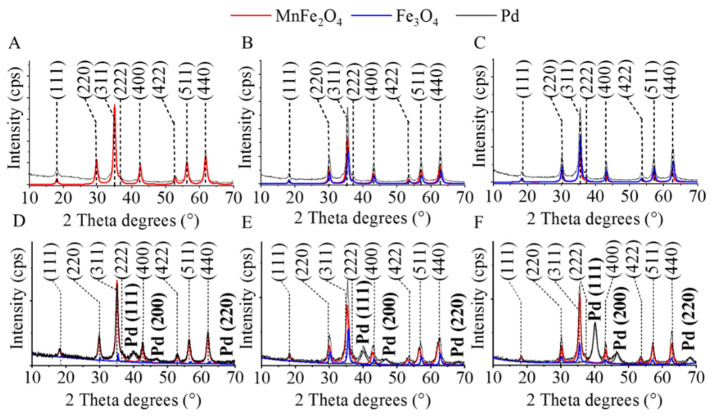
XRD diffractograms of the magnetic catalyst supports prepared at 573 K (**A**), 623 K (**B**), and 673 K (**C**) and the corresponding palladium-decorated catalysts (**D**) 573 K, (**E**) 623 K, and (**F**) 673 K.

For the identification and quantification of the different crystalline phases in the magnetic catalyst supports and the palladium-decorated catalysts, XRD analysis was performed (Figure 3). On the diffractograms of the magnetic ferrite samples (Figure 3A), reflexions at 18.1°, 29.9°, 35.3°, 36.8°, 42.5°, 52.7°, 56.3°, and 61.7° two Theta degrees were identified, which correspond to (111), (220), (311), (222), (400), (422), (511), and (440) Miller-indexed crystal lattices of the MnFe_2_O_4_ phase (PDF 74–2403), respectively. In the case of the ferrite sample, which was prepared at 573 K, only one phase, manganese ferrite, was found (Figure 3A). On the diffractograms of the magnetic nanopowders, which were made at 623 K and 673 K, magnetite (Fe_3_O_4_) was also identified, in addition to the Mn spinel (Figure 3B,C). The reflexions, which are characteristic for the Fe_3_O_4_ are identified at 18.2° (111), 30.3° (220), 35.7° (311), 43.2° (400), 53.5° (422), 57.1° (511), and 62.3° (440) two Theta degrees (PDF 19-629).

Surprisingly, magnetite was also identified in the pure manganese ferrite catalyst support in the case of the palladium-decorated magnetic catalysts. (Figure 3D). This phenomenon can be explained by the decomposition of the spinel upon sonication and the subsequent phase-separation of magnetite. This phase transition could be promoted by the high energy released during the acoustic cavitation, which was applied for the palladium decomposition on the surface of the MnFe_2_O_4_ particles. Magnetite was also found in the other two catalysts (Figure 3E,F). The reflexions of the elemental palladium are located at 40.2°, 46.3°, and 68.0° two Theta degrees which belong to the Pd (111), Pd (200), and Pd (220) phases (PDF 046–1043), respectively.

Based on the Rietveld analysis, only MnFe_2_O_4_ (maximum spinel phase) was identified in the sample prepared at the lowest temperature (573 K, Table 1). The average particle size was 11 ± 3 nm. By increasing the temperature to 623 K and 673 K during the heating step, the magnetite phase also formed in increasing amounts (38.9 wt% and 48.2 wt%, respectively) in addition to the manganese spinel. The particle sizes were similar in the case of the ferrite and magnetite crystallites (between 11 nm and 14 nm). The deposition of the palladium particles onto surface of the magnetic crystallites was carried out by high-energy ultrasonic treatment. Due to the high energy, the manganese ferrite (jacobsite) partially decomposed, and magnetite formed. Nevertheless, this does not affect separability since the nanopowders contain only magnetic phases in addition to metallic palladium. The average crystallite size of the palladium nanoparticles is between 4 nm and 6 nm. The real palladium contents were determined by ICP-OES measurements, and the results were similar (between 4.20 and 4.65 wt%) for the three catalysts (Table 1).

Sorptometric investigations revealed no significant differences (approx. 10%) between the specific surface areas (A_DA_, Table 1) of the palladium-containing magnetic catalysts prepared at different temperatures. It was found that, by increasing the synthesis temperature from 573 to 623 K, the surface is slightly decreased (74 m^2^ g^−1^ for Pd/MnFe_2_O_4_-573 K and 69 m^2^ g^−1^ for Pd/MnFe_2_O_4_-623 K, respectively). However, if the temperature is further increased up to 673 K, the surface area also increases and it becomes even larger than at 573 K (78 m^2^ g^−1^, Pd/MnFe_2_O_4_-673 K).

### 2.2. Catalytic Tests of the Prepared Magnetic Catalysts in Nitrobenzene Hydrogenation

**Figure 4:**
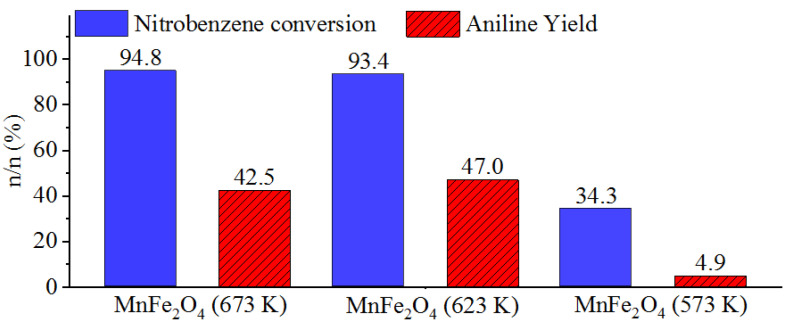
Nitrobenzene conversions and aniline yields achieved by using the palladium-free MnFe_2_O_4_ supports prepared at 673 K, 623 K, and 573 K. (T = 323 K, *p* = 20 bar H_2_).

To check the catalytic activity of the supports, the palladium-free manganese ferrite samples were tested in nitrobenzene hydrogenation at 323 K temperature and 20 bar hydrogen pressure. All the samples turned out to be catalytically active. The highest nitrobenzene conversion (94.8 n/n%) was reached by using the MnFe_2_O_4_ sample prepared at 673 K. However, the aniline yield was only 42.5 n/n% (Figure 4, Table 1). Similar results were achieved by applying the other support prepared at 623 K, but in this case, the nitrobenzene conversion was slightly lower (93.4 n/n%), while the aniline yield was a bit higher, at 47.0 n/n%. In the case of the MnFe_2_O_4_ prepared at 573 K, the nitrobenzene conversion and aniline yield were only 34.3 n/n% and 4.9 n/n%, respectively (Table 1).

Despite the high (X > 90%) nitrobenzene conversions in two cases, the corresponding aniline yields (Y < 50%) were unsatisfactory, and thus, the application of palladium is necessary.

**Figure 5:**
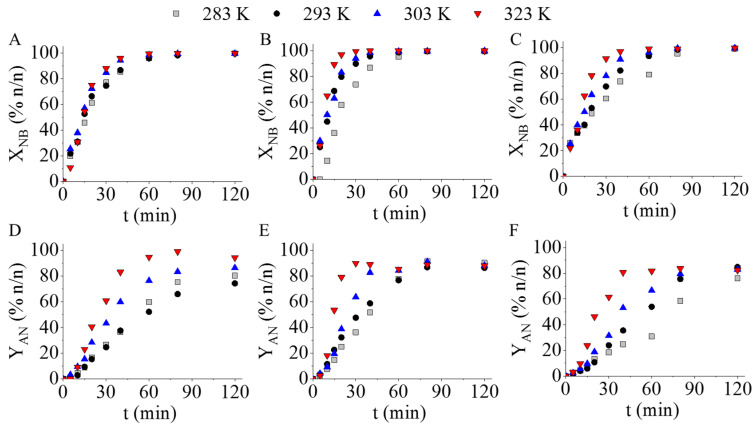
Nitrobenzene conversion and aniline yield as a function of hydrogenation time by using the magnetic Pd catalysts (Pd/MnFe_2_O_4_) prepared at 573 K (**A**,**D**), 623 K (**B**,**E**), and 673 K (**C**,**F**). The different marks represent different reaction temperatures.

Next, the novel palladium-decorated ferrite nanoparticle catalysts were tested in nitrobenzene hydrogenation at four different reaction temperatures (283 K, 293 K, 303 K, and 323 K) and at constant hydrogen pressure (20 bar). Almost complete nitrobenzene conversion (99.9 n/n%) was reached for all three Pd-decorated catalysts; however, the reaction time varied significantly. For Pd/MnFe_2_O_4_ (623 K) it took only 40 min to reach complete conversion, while in the case of Pd/MnFe_2_O_4_ (573 K) and Pd/MnFe_2_O_4_ (673 K) 180 min and 120 min, respectively, were necessary (Figure 5A, and Table 1).

The change of reaction temperature does not have a significant effect on the nitrobenzene conversions (X_NB_), but it does affect the aniline yield (Y_AN_). The highest yield (96.8 n/n%) was achieved after 240 min hydrogenation at 283 K in the case of the Pd/MnFe_2_O_4_ (623 K) catalyst. The formation of aniline from its intermediates (azobenzene and azoxybenzene) showed a slowing trend at lower temperatures. In contrast to the catalyst prepared at 573 K, in the case of the two other catalysts, Pd/MnFe_2_O_4_ (623 K) and Pd/MnFe_2_O_4_ (673 K), reaction temperature had a more pronounced effect on the nitrobenzene conversion. This may be explained by the significantly different magnetite loading of the three catalysts, namely the Pd/MnFe_2_O_4_ (573 K) contained only 3.3 wt% Fe_3_O_4_ phase, while the two other samples contained 25.7 wt% and 20.3 wt% (Table 1). The corresponding palladium-free manganese ferrites (38.9 wt%, 623 K and 48.2 wt%, 673 K magnetite content) showed higher catalytic activity than the pure MnFe_2_O_4_-523 K (Figure 4). In summary, the different magnetite content may be able to influence the catalytic activity and temperature sensitivity of the tested palladium-decorated ferrite catalysts.

**Figure 6:**
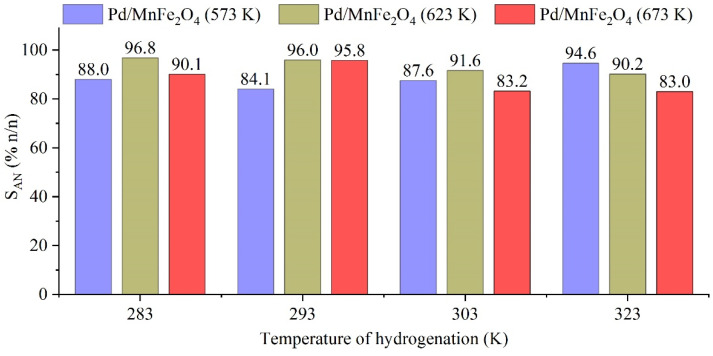
Aniline selectivity vs. hydrogenation temperature by using the prepared Pd/MnFe_2_O_4_ (573 K), Pd/MnFe_2_O_4_ (623 K), and Pd/MnFe_2_O_4_ (673 K) catalysts.

The maximum aniline selectivity (S_AN_) versus reaction temperature was also determined in the case of the three Pd/MnFe_2_O_4_ catalysts (Figure 6). High selectivity values were obtained for each catalyst/reaction temperature pairs. At 283 K, the Pd/MnFe_2_O_4_ (623 K) catalyst showed the highest selectivity. Common by-products (e.g., *N*-methylaniline, or *o*-toluidine) were not detected. During the reaction, the presence of intermediates, azobenzene and azoxybenzene, were confirmed, but these were completely converted to aniline until the end of the reaction. At lower temperatures (283 K and 293 K), the aniline selectivity was S_AN_ > 96 n/n%. There were no significant amounts of by-products detected during the catalytic test; therefore, the selectivity lower than 100% may be explained by the formation of polyaniline, which could not be detected by GC–MS measurements. In all, the prepared manganese ferrite–supported palladium catalysts are highly selective and may be applicable in aniline synthesis.

**Figure 7:**
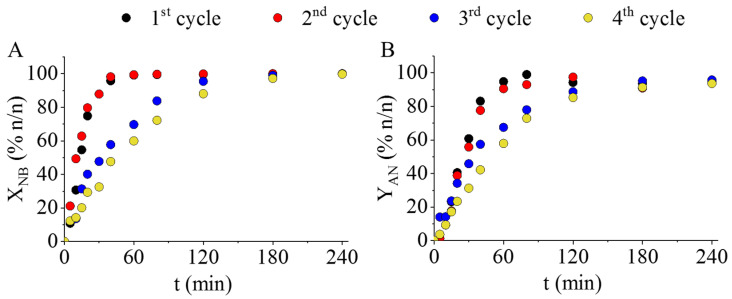
Reuse tests of the Pd/MnFe_2_O_4_ (573 K) catalyst. Nitrobenzene conversion (**A**) and aniline yield (**B**) vs. time of hydrogenation during the catalytic tests.

From the three developed magnetic catalysts, the one which was prepared at 573 K was selected to carry out reuse tests because its activity was less sensitive to the hydrogenation temperature, which may be related to the very low magnetite content. The reuse tests were carried at four cycles at 323 K. The nitrobenzene conversion and aniline yield did not change significantly during the time (180 min) of hydrogenation (Figure 7A,B).

During the third and fourth reuse tests, nitrobenzene conversion turned out to be lower during the initial phase of hydrogenation. However, after 180 min reaction time, the conversion and yield values were the same as those of the first two cycles. That is, the catalysts were able to convert the total amount of nitrobenzene with maximum aniline yield in each cycle. It should be noted, however, that the catalyst was not regenerated between the cycles, it was only washed with methanol. Thus, the developed Pd/MnFe_2_O_4_ catalyst is applicable in at least in four cycles of hydrogenation without regeneration. In addition, the magnetic feature of the catalyst further increases its applicability in the above commercially important hydrogenation system.

## 3. Materials and Methods

The spinel nanopowders were made from manganese(II) nitrate tetrahydrate (Mn(NO_3_)_2_∙4H_2_O, MW: 251.01 g/mol, Carl Roth GmbH, D-76185 Karlsruhe, Germany), iron(III) nitrate nonahydrate (Fe(NO_3_)_3_∙9H_2_O, MW: 404.0 g/mol, VWR Int. Ltd., B-3001 Leuven, Belgium). As reducing agent and dispersion medium, polyethylene glycol (PEG 400, Mw: ~400 Da, which corresponds to a chain length of approximately 8–9 links) from VWR Int. Ltd. (F-94126 Fontenay-sous-Bois, France), was applied. Palladium(II) nitrate dihydrate (Pd(NO_3_)_2_*2H_2_O, MW: 266.46 g/mol, Thermo Fisher Ltd., D-76870 Kandel, Germany) as Pd precursor and Patosolv^®^, a mixture of 90 vol% ethanol and 10 vol% isopropanol (Molar Chem. Ltd., H-2314 Halásztelek, Hungary) were used during the preparation of the magnetic catalyst. Catalytic tests were carried out on nitrobenzene (NB, Merck KGaA, D-64293 Darmstadt, Germany).

### 3.1. Preparation of the Magnetic Spinel Nanoparticles and the Final Pd Catalyst

Manganese ferrite magnetic catalyst supports were synthesized in a two-step process, involving a sonochemical and a heat treatment step in air (Figure 1). In the sonochemical step, 6.44 g (15.9 mmol) iron(III) nitrate nonahydrate and 2.00 g (7.97 mmol) manganese(II) nitrate tetrahydrate were dissolved in 20 g polyethylene glycol, and the solution was sonicated by using a Hielscher UIP1000 Hdt homogenizer for 3 min (120 W, 17 kHz). The energy released during ultrasonic cavitation was enough to support the formation of metal oxyhydroxide nanoparticles from the metal precursors. The reddish-brown viscous dispersions were heated for 3 h at 573 K, 623 K, and 673 K, respectively. After elimination or burning of the polyethylene glycol, dehydration of the oxide-hydroxides occurred, resulting in the formation of a spinel phase. For the deposition of the palladium nanoparticles onto the surface of the ferrite crystals, a Hielscher UIP100 Hdt homogenizer was used. First, palladium(II) nitrate (0.25 g, 0.94 mmol) was dissolved in patosolv (50 mL) within which ferrite sample (2.00 g) was dispersed and then was treated by ultrasonic cavitation for two minutes.

### 3.2. Catalytic Tests—Nitrobenzene Hydrogenation

0.10 g magnetic spinel supported Pd catalysts were used for nitrobenzene (NB, c = 0.25 mol/dm^3^ in methanolic solution) hydrogenation in an agitated (1000 rpm), Büchi Uster Picoclave reactor of 200 mL volume. The pressure of the hydrogenation was kept at 20 bar in all experiments and the reaction temperature was set to 283 K, 293 K, 303 K, and 323 K. Samples for analysis were taken after 0, 5, 10, 15, 20, 30, 40, 60, 80, 120, 180, and 240 min of hydrogenation.

### 3.3. Characterization Techniques

High-resolution transmission electron microscopy (HRTEM, FEI Technai G2 electron microscope, 200 kV) was used for size and morphological characterization of the nanoparticles. During sample preparation an aqueous suspension of the nanoparticles was dropped on 300 mesh copper grids (Ted Pella Inc., Redding, CA, USA). The qualitative and the quantitative analysis of the different oxide forms was carried out with X-ray diffraction (XRD) measurements by applying the Rietveld method. Bruker D8 diffractometer (Cu-Kα source) in parallel beam geometry (Göbel mirror) with Vantec detector was used. The carbon content, which remained from the polyol, was determined by Vario Macro CHNS element analyzer for all ferrite samples. Phenanthrene was used as standard (C: 93.538%, H: 5.629%, N:0.179%, S: 0.453%) from Carlo Erba Inc. Helium (99.9990%) was the carrier gas, and oxygen (99.995%) was used as oxidative atmosphere. Electrokinetic (Zeta) potential measurements were performed on the ferrite samples by using their aqueous colloids with Malvern Zetasizer Nano ZS equipment. The Zeta potentials were calculated based on electrophoretic mobility measurements by applying laser Doppler electrophoresis. The functional groups located on the surface of the prepared nanopowders were identified with Fourier transform infrared spectroscopy (FTIR) by using a Bruker Vertex 70 spectroscope. The measurements were carried out in transmission mode, and in each case, a 10 mg sample was pelletized with 250 mg potassium bromide. The palladium contents of the catalyst samples were determined by using a Varian 720 ES inductively coupled optical emission spectrometer (ICP-OES). For the ICP-OES measurements the samples were dissolved in aqua regia. The specific surface area of the catalysts was measured by CO_2_ adsorption–desorption experiments at 273 K by using a Micromeritics ASAP 2020 sorptometer based on the Dubinin–Astakhov (DA) method. After the hydrogenation tests, the aniline-containing samples were quantitatively analyzed by using an Agilent 7890A gas chromatograph coupled with Agilent 5975C Mass Selective detector. An RTX-624 column (60 m × 0.25 mm × 1.4 μm) was used and the injected sample volume was 1 μL at 200:1 split ratio, while the inlet temperature was set to 473 K. Helium was used as the carrier gas (2.28 mL/min), and the oven temperature was set to 323 K for 3 min and it was heated up to 523 K with a heating rate of 10 K/min and kept there for another 3 min. The analytical standards of aniline, potential by-products, and the intermediates originated from Sigma Aldrich (Burlington, MA, USA) and Dr. Ehrenstorfer Ltd. (Wesel, Nordrhein, Germany).

The catalytic activity of the magnetic Pd catalysts was compared based on Equation (1):

(1) $$Y=X\times S=\frac{\;(n_{NB,t}-n_{NB,0})}{n_{NB,0}}\times \frac{n_{AN,t}}{\;(n_{NB,t}-n_{NB,0})}\;$$

where *Y* is the yield of aniline, *X* the conversion of nitrobenzene, *S* aniline selectivity, $n_{NB,\;0}$ the initial amount of nitrobenzene at *t* = 0, $\left(n_{NB,\;t}-n_{NB,\;0}\right)$ the amount of nitrobenzene consumed after t reaction time and $n_{AN,t}$ the amount of aniline formed. In the main text the % values are presented, which are obtained by multiplying *Y*, *X*, and *S* by 100.

## 4. Conclusions

Manganese ferrite–supported magnetic separable palladium catalysts (Pd/MnFe_2_O_4_) were prepared using a simple sonochemical method starting from the corresponding metal nitrates and consecutive heat treatment at three different temperatures of the formed metal-(oxi)hydroxides. Each of these catalysts shows high catalytic activity in nitrobenzene hydrogenation even without the noble metal (Pd). Catalytic activity investigations of the Pd-free magnetic supports revealed that the noble metal–free system can reach up to 94% nitrobenzene conversion. Due to the local hotspots during sonication, magnetite also formed outside of the ferrite phase, which proved to be crucial for increased catalytic activity, since without magnetite only low (<34 n/n%) conversions were determined. However, the aniline yield was unsatisfactory in each case for the pure supports, and thus, the inclusion of palladium was inevitable. The catalytic activity of the two nanomaterials (MnFe_2_O_4_ and Pd) combined led to a fast, efficient, and selective catalyst. In addition to the nitrobenzene conversion and aniline yield, the aniline selectivity of the catalysts is also remarkable (above 96 n/n%). The increasing amount of magnetite in addition to manganese ferrite greatly affects the temperature sensitivity of the nitrobenzene conversion. The Pd/MnFe_2_O_4_ (573 K) catalyst’s activity had the lowest sensitivity for the hydrogenation temperature, which may be related to its very low magnetite content. Reuse tests showed that the catalyst prepared at 573 K can give the same performance in at least four cycles without regeneration. The ease of preparation, combined with high conversion, yield and selectivity, as well as the reusability without regeneration, make the developed Pd/MnFe_2_O_4_ magnetic catalyst system successfully applicable in the industrially important hydrogenation of nitro compounds.

## Figures and Tables

**Table 1:** Summary table for the physical and chemical properties of the magnetic nanoparticles prepared: mean particle sizes and the phase composition of the manganese ferrite and magnetite based on XRD results, the palladium contents based on ICP-OES analysis, **A_DA_** specific surface area determined by the Dubinin–Astakhov (DA) method. **X_max_**, **Y_max_** and **S_max_** are the maximum conversion, yield, and selectivity values determined at the time and temperature indicated in parentheses.

	MnFe_2_O_4_		Fe_3_O_4_		Pd		A_DA_ m^2^/g	X_max,_ (t) (%)	Y_max._(t) (%)	S_max._(t) (%)
	d (nm)	wt%	d (nm)	wt%	d (nm)	wt%				
MnFe_2_O_4_ (573 K)	11 ± 3	100	ᴓ	ᴓ	ᴓ	ᴓ	ᴓ	34.3 ^a^	4.90 ^a^	n/a
MnFe_2_O_4_ (623 K)	12 ± 2	61.1	14 ± 3	38.9	ᴓ	ᴓ	ᴓ	93.4 ^a^	47.0 ^a^	n/a
MnFe_2_O_4_ (673 K)	13 ± 2	41.8	12 ± 3	48.2	ᴓ	ᴓ	ᴓ	94.8 ^a^	42.5 ^a^	n/a
Pd/MnFe_2_O_4_ (573 K)	10 ± 3	89.1	35 ± 5	3.3	4 ± 1	4.20	74	99.9 (180 min) (303 K)	94.8 (60 min) (323 K)	94.6 (120 min) (323 K)
Pd/MnFe_2_O_4_ (623 K)	8 ± 2	66.0	15 ± 3	25.7	4 ± 1	4.64	69	99.9 (40 min) (323 K)	96.7 (240 min) (283 K)	96.8 (240 min) (283 K)
Pd/MnFe_2_O_4_ (673 K)	10 ± 3	64.3	13 ± 2	20.3	6 ± 1	4.61	78	99.9 (120 min) (323 K)	95.7 (180 min) (293 K)	95.8 (180 min) (293K)

**^a^** These parameters were measured at 240 min and 323 K.

## Data Availability

Data is available upon request from the corresponding authors.
